# A Case Report on a Paratesticular Tumor Mass: A Myxoid Spindle Cell Neoplasm With Angiomyxoma Features

**DOI:** 10.7759/cureus.82686

**Published:** 2025-04-21

**Authors:** Shani M Abraham, Linda Gavric, Dencie Devora, M. Rudwan Soukieh, Michael W Fountain

**Affiliations:** 1 Internal Medicine, Lake Erie College of Osteopathic Medicine, Bradenton, USA; 2 Psychiatry and Behavioral Sciences, Lake Erie College of Osteopathic Medicine, Bradenton, USA; 3 College of Medicine, Lake Erie College of Osteopathic Medicine, Bradenton, USA; 4 Urology, AdventHealth, Tavares, USA

**Keywords:** angiomyxoma, epididymis, myxoid neoplasm, paratesticular tumor, surgery, testicle

## Abstract

This case report presents a 53-year-old male patient complaining of left-sided testicular pain. Upon scrotal ultrasound, the presence of a paratesticular tumor was determined. Upon further surgical exploration and tissue examination, the lesion was determined to be a myxoid spindle cell neoplasm with angiomyxoma features.

This case report analyzes this benign lesion and provides an appropriate treatment plan, including surgery and imaging for follow-up. Paratesticular tumors are rare findings, and there is a lack of research on optimal treatment plans for these rare cases. The case report adds to the literature by increasing our understanding of this rare testicular tumor and providing an appropriate treatment plan to consider and utilize when treating these rare cases.

## Introduction

Paratesticular tumors are very rare tumors, with an incidence of 5% or less and a prevalence of 3-6% [1]. Since these tumors are infrequent, it is vital to understand how to approach assessment and treatment when they arise. The paratesticular anatomy is defined as the structures surrounding the testis, including the epididymis, spermatic cord, and tunica vaginalis [2]. These tumors arise in this region and may range from benign to locally invasive to metastatic. In this case report, a male patient presented with left-sided testicular pain and underwent a scrotal ultrasound, which revealed the presence of a possible paratesticular tumor. Upon surgical exploration and tissue examination, a myxoid spindle cell tumor with angiomyxoma features was noted.

Through this case report, we seek to add to the literature a better understanding of this specific form of rare paratesticular tumor. We also hope to increase awareness and understanding of a treatment plan for this rare case, providing rationale, based on the few previous studies available, as well as the treatment plan used in this specific case.

## Case presentation

**Figure 1:**
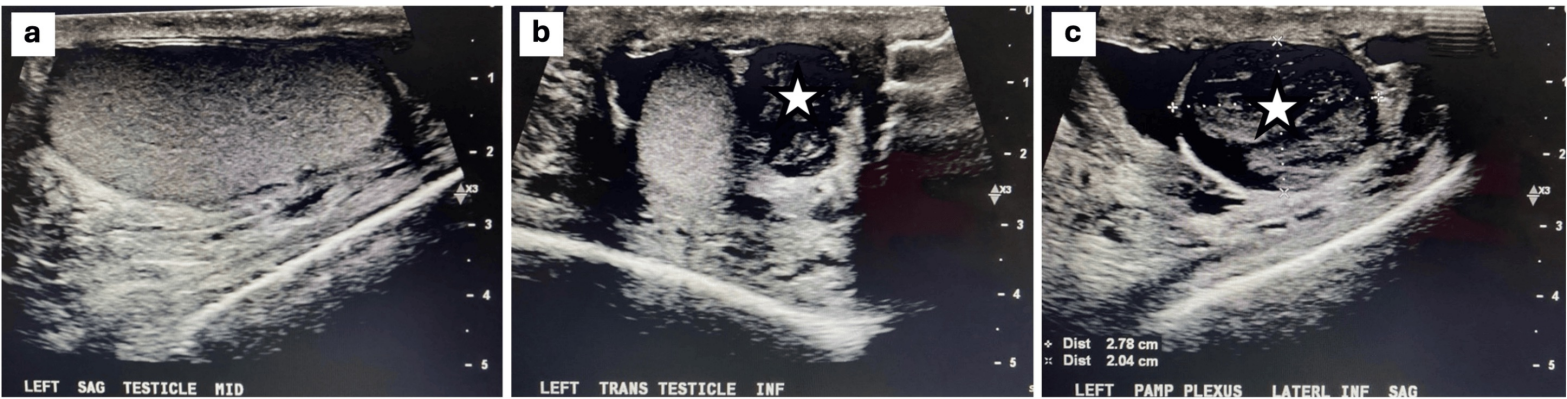
Scrotal ultrasound images upon presentation. (a) Sagittal mid view of the left testicle. (b) Transverse view of the left testicle and supratesticular mass. (c) Lateral inferior sagittal view of the left pampiniform plexus. This view also demonstrates the supratesticular mass. The star represents the supratesticular mass. All scale bars represent centimeters.

A 53-year-old man presented with testicular pain on the left side. A scrotal ultrasound was completed and demonstrated a suspicious heterogenous structure within the left epididymis as well as a small hydrocele and varicocele on the left side. Upon result review with the Urology team, it was suspected that this suspicious supratesticular mass on the scrotal ultrasound could be a possible growing adenomatoid tumor. A plan was set to move forward with surgical exploration to remove the supratesticular mass, as well as possible orchiectomy on the left side. The scrotal ultrasound demonstrated the testicular mass to be 2.8 x 2.8 x 2.0 cm in dimension. Figure 1a shows the sagittal mid-view of the left testicle. Figure 1b shows the transverse view of the left testicle and supratesticular mass. Figure 1c shows the lateral inferior sagittal view of the left pampiniform plexus. This view also demonstrates the supratesticular mass.

Prior to the surgical procedure, the patient’s past medical history was reviewed and included smoking, EtOH use, umbilical hernia, and snoring. The patient had a body mass index of 31.4. Labs were ordered and reviewed prior to the procedure to ensure patient safety. A complete blood count (CBC) (Table 1), complete metabolic panel (CMP) (Table 2), and urine culture (Table 3) were completed by the patient, and the values were within normal limits to proceed with the surgical exploration procedure. The risks and benefits of the procedure were discussed at length with the patient, and the decision was made to move forward with the excision of the mass.

**Table 1:** Complete blood count

Complete blood cell count	Reference Range	Value
White Blood Cell	3.8-10.8 thousand/uL	6.5
Red Blood Cell	4.2-5.8 million/uL	4.92
Hemoglobin	13.2-17.1 g/dL	16.7
Hematocrit	38.5%-50%	49.1%
Mean Corpuscular Volume	80-100 fl	99.8
Mean Corpuscular Hemoglobin	27-33 pg	33.9
Mean Corpuscular Hemoglobin Concentration	32-36 g/dL	34
Red Cell Distribution Width	11%-15%	12.5%
Platelet Count	140-400 thousand/uL	208
Mean Platelet Volume	7.5-12.5 fL	10.6
Neutrophil abs	1500-7800 cells/uL	3835
Lymphocyte abs	850-3900 cells/uL	2054
Monocyte abs	200-950 cells/uL	462
Eosinophils abs	15-500 cells/uL	117
Basophils abs	0-200 cells/uL	33
Neutrophil %	Not applicable	59.0
Lymphocyte %	Not applicable	31.6
Monocyte %	Not applicable	7.1
Eosinophil %	Not applicable	1.8
Basophil %	Not applicable	0.5

**Table 2:** Comprehensive metabolic panel

Complete Metabolic Panel	Reference Range	Value
Glucose	65-99	91
Blood Urea Nitrogen	7-25 mg/dL	8
Creatinine	0.7-1.3 mg/dL	0.84
eGFR	Greater than or equal to 0.60 ml/min/1.73 m^2	106
Na	135-146 mmol/L	136
K	3.5-5.3 mmol/L	4.6
Cl	98-100 mmol/L	99
CO2	20-32 mmol/L	26
Ca	8.6-10.3 mg/dL	10

**Table 3:** Urine culture

Test status	Final
Specimen source	Urine, clean catch
Specimen quality	Adequate
Result	No growth

**Figure 2:**
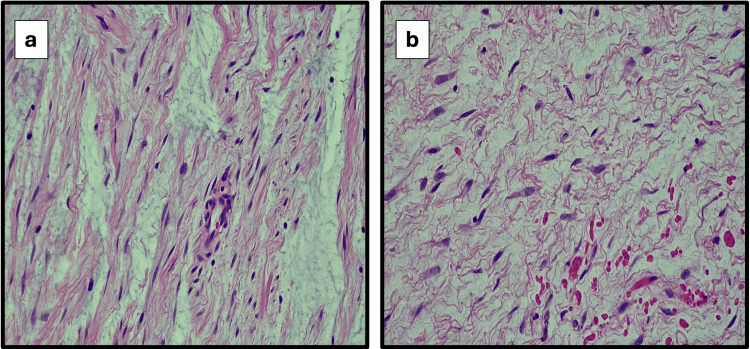
Myxoid spindle cell neoplasm with features of angiomyxoma 2a and 2b represent a myxoid spindle cell neoplasm with features of angiomyxoma. Hematoxylin and eosin stain was used. The magnification of the images is 400x (40x objective lens and 10x eyepiece).

Operatively, the patient underwent induction of general anesthesia, and the left lateral scrotum was injected with topical anesthesia. An incision was made, and a sharp and blunt dissection was used to localize the mass. The mass was noted to be present between the testicle and the head of the epididymis. The mass was dissected free of surrounding structures without identification of inflammatory changes. The area was irrigated and then closed in multiple layers. The mass was excised completely and measured 3.0 x 3.0 x 2.5 cm in dimension. The tissue was sent to be examined, and the results revealed a myxoid spindle cell neoplasm with features of angiomyxoma. Figures 2a, 2b represent the histology of the myxoid spindle cell neoplasm. Hematoxylin and eosin stain was used. Magnification of the images is 400x (40x objective lens and 10x eyepiece).

Table 4 further clarifies the results of the paratesticular tumor test.

**Table 4:** Immunohistochemistry panel Results depicted CD34 and CDK4 as variably positive and P16 as uniformly positive.

Microscopic Description	Result
OSCAR	Negative
S100	Negative
SOX10	Negative
NF	Negative
CD34	Variably positive
Beta-catenin	Negative for nuclear
SMA	Negative
Desmin	Negative
MUC4	Negative
STAT6	Negative
P16	Uniformly positive
MDM2	Negative
CDK4	Variably positive
RB1	No loss of expression
Ki67	Approximately 5%

The immunohistochemistry panel helped rule out different forms of neoplasms and determine the specific form of neoplasm. A fibrous tumor was ruled out since STAT6 was negative, fibromyxoid sarcoma since MUC4 was negative, fibromatosis since beta-catenin was negative, epithelial neoplasm since OSCAR was negative, neurogenic neoplasm since S100/SOX10 was negative, and myogenic neoplasm since SMA and desmin were negative.

To rule out a possible liposarcoma, further testing with fluorescence in situ hybridization (FISH) analysis was completed with a DDIT3 (CHOP) probe and MDM2 probe, and the results were negative. The final result was a myxoid spindle cell neoplasm, with features of angiomyxoma.

At a follow-up encounter with the patient, tissue type was disclosed, and no complications were reported. A repeat scrotal ultrasound was ordered to be completed within three to six months of mass excision. Since the tissue was revealed to be presumptively benign, observation through ultrasound was determined to be the follow-up plan. This case was also reviewed with a Hematology-Oncology consultant.

**Figure 3:**
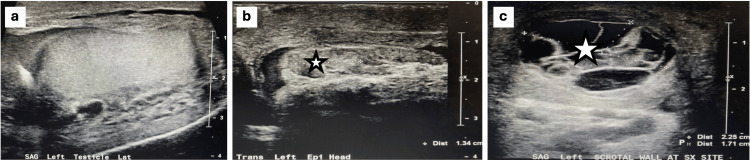
Scrotal ultrasound images upon presentation (a) Lateral left testicle. (b) Trans view of left epididymis head. (c) Left scrotal wall view at surgery site. The star represents the surgical site. All scale bars represent centimeters.

However, four weeks post-procedure, the patient noted a possible new mass present below the initial incision from the scrotal surgery. An ultrasound was completed earlier than initially planned and revealed normal left and right testicles and epididymis. The test also revealed a complex cystic lesion present in the midline of the incision from the previous operation. The lesion measured about 2.3 cm in dimension and represented a probable hematoma post-surgical intervention. This was a benign finding. See Figure 3a representing the left lateral testicle. See Figure 3b representing the left epididymis head transverse view. See Figure 3c representing the left scrotal wall view at the surgery site.

Due to the patient having a history of myxoid spindle cell neoplasm with features of angiomyxoma, the possibility of a cystic neoplasm is not to be excluded. Regarding further testing and follow-up, another scrotal ultrasound is planned to be completed in the following four weeks to confirm a decrease in the size of the lesion.

## Discussion

Paratesticular tumors refer to tumors in the paratesticular space that do not arise from the testicular parenchyma. This space includes the epididymis, tunica vaginalis, and the spermatic cord [2]. When assessing abnormalities within the testicle and paratesticular space, ultrasound is often used as the initial imaging modality; however, it may be nonspecific [2]. A majority of paratesticular tumors are benign, but about 30% are malignant, with sarcomas making up 90% of the malignant cases [3]. About 70% of paratesticular tumors are present in the spermatic cord, and the most common form of tumor is a benign lipoma. It is vital to consider the different possibilities when assessing a paratesticular lesion, including benign and malignant forms. Malignant entities are mostly sarcomas since the spermatic cord is derived from the mesoderm, with rhabdomyosarcoma and liposarcoma being the most common [2]. Liposarcoma is more common in older adults (peak age of 56) while rhabdomyosarcoma is more common in children (peak age of 5 years, and another peak at 16 years) [2]. Another important malignant entity to consider is lymphoma, which typically and primarily appears within and around the testicle, but it may also impact the epididymis and spermatic cord. Primary testicular lymphoma was vital to consider in the case of this patient, as he presented with an abnormal mass at the age of 53. Primary testicular cancer is more common in male patients in their early 30s, with an average age of 33 [4]. However, after 50 years of age, cancer in the testicle region is more commonly due to secondary testicular cancer (primary testicular lymphoma) rather than primary testicular cancer [5].

Benign entities of paratesticular lesions to consider include hydroceles due to fluid accumulation within the tunica vaginalis, and inflammatory lesions such as epididymitis [2]. Inflammatory causes can differ depending on the age of the patient. For example, Chlamydia and Neisseria gonorrhoeae are more common causes of epididymitis in younger men, and Escherichia coli is the more common cause of epididymitis in older men [2]. Other causes of benign lesions in the paratesticular space include spermatic cord and epididymal cysts (presenting with serous fluid), lipoma (presenting with fat lobules), leiomyoma (presenting with smooth muscle), and hemangioma (different types including capillary, arteriovenous, and cavernous) [2]. Regarding this case and patient, when the initial ultrasound was completed, the possibility of the scrotal mass being an adenomatoid tumor was suggested. Adenomatoid tumors make up about 30% of paratesticular masses and are mesothelial tumors [6]. Adenomatoid tumors are benign tumors of the genital tract, and more commonly occur in white males with an age range of 30-50 years [6]. In males, these tumors are more likely to occur within the epididymis, prostate, spermatic cord, or ejaculatory duct. They appear on ultrasound as homogenous, hyper, or hypoechoic and solid [6]. Although the mass assessed on ultrasound was suspicious for an adenomatoid tumor, upon surgical excision and tissue exam results, it was determined that the mass was a myxoid spindle cell neoplasm with features of angiomyxoma.

Myxoid soft tissue tumors are uncommon, heterogeneous tumors composed of extracellular myxoid matrix. The matrix is made up largely of water, gelatinous glycosaminoglycans, and mucopolysaccharides [7]. Myxoid tumors can be classified through features of MRI, with T1 showing a hypointense enhancing lesion, and T2 showing a bright enhancing lesion. Myxoid soft tissue tumors can range in classification from benign to locally invasive to malignant. The benign classifications include but are not limited to intramuscular myxoma, angiomyxoma, myxolipoma, and fibromyxoma. Our patient presented with the pathological result of a myxoid spindle cell tumor with features of angiomyxoma, directing us to believe the mass is benign. The malignant classification of myxoid tumors includes but is not limited to liposarcoma, chondrosarcoma, and myxofibrosarcoma [7]. The most common form of benign myxoid neoplasm is an intramuscular myxoma of the thigh. Myxoid neoplasms commonly affect the extremities over visceral organs [7]. In this case, the patient presented with a myxoid mass characterized by the presence of spindle cells. Spindle cells refer to the shape of the tumor cells, being long and thin, with a cigar-shaped nucleus in the center [8].

The patient also presented with a feature of angiomyxoma. Angiomyxoma is further defined as a rare soft tissue mass (myxoma) with extensive blood vessels that grows slowly and does not spread [9]. Angiomyxomas grow from myxoid cells found in connective tissue. Angiomyxomas can be further separated as superficial or aggressive. The superficial form is either on the skin or right below the surface. The superficial form may grow on the abdominopelvic, cervical, and extremity regions and are more common in older adults [9]. The aggressive form of angiomyxoma grows deeper but is still unlikely to metastasize. The aggressive form grows more commonly in the perineum and pelvic region and is more common in women [9]. In a study by Calonje et al., 39 cases of superficial angiomyxoma were assessed [10]. In the study, the age range of patients was a median of 45.5 years, ranging from birth to 82 years. Most of the superficial angiomyxomas arose from the trunk, then the head/neck, and then the lower limbs. All 39 cases had excision of the mass as the primary treatment [10]. Histologic features from cases included myxoid stroma, blood vessels, varied cellularity, and fibroblastic cells. Regarding aggressive angiomyxoma, Sutton et al. noted that aggressive angiomyxomas are frequently initially misdiagnosed as lipomas, hernias, or cysts [11]. The aggressive forms may be positive for estrogen and progesterone receptors. Surgical excision is the best form of treatment, but gonadotropic-releasing hormone agonist is another treatment of choice to consider [11]. Based on our patient’s histology report, the mass excised is better characterized as one with superficial angiomyxoma features.

## Conclusions

This case depicts a 53-year-old male with a rare paratesticular tumor, further specified as a myxoid spindle cell neoplasm with angiomyxoma features. This case is unique because paratesticular tumors in general are very rare. This case was meant to reveal a greater level of understanding of this specific form of benign tumor and demonstrate an appropriate treatment plan. The main treatment for this patient was surgical resection. The follow-up plan was to monitor the progression of the lesion post-removal of the mass with scrotal ultrasound. Imaging and follow-up with the patient at set months post-procedure are essential to detect possible recurrence or metastasis. In this case, we planned to reassess with a scrotal ultrasound within three to six months. We would like to encourage more case reports with an emphasis on the timeline of paratesticular tumors regarding treatment/follow-up and possible recurrences. By increasing research in this area, we will be able to better determine the optimal form of treatment and create guidelines for these rare cases that arise.
